# Activating Transcription Factor 3 (ATF3) is a Highly Conserved Pro-regenerative Transcription Factor in the Vertebrate Nervous System

**DOI:** 10.3389/fcell.2022.824036

**Published:** 2022-03-08

**Authors:** Hilary R. Katz, Anthony A. Arcese, Ona Bloom, Jennifer R. Morgan

**Affiliations:** ^1^ The Eugene Bell Center for Regenerative Biology and Tissue Engineering, Marine Biological Laboratory, Woods Hole, MA, United States; ^2^ The Feinstein Institutes for Medical Research, Manhasset, NY, United States; ^3^ The Donald and Barbara Zucker School of Medicine, Hempstead, NY, United States

**Keywords:** regeneration, spinal cord injury, zebrafish, lamprey, dorsal root ganglia (DRG) neurons

## Abstract

The vertebrate nervous system exhibits dramatic variability in regenerative capacity across species and neuronal populations. For example, while the mammalian central nervous system (CNS) is limited in its regenerative capacity, the CNS of many other vertebrates readily regenerates after injury, as does the peripheral nervous system (PNS) of mammals. Comparing molecular responses across species and tissues can therefore provide valuable insights into both conserved and distinct mechanisms of successful regeneration. One gene that is emerging as a conserved pro-regenerative factor across vertebrates is activating transcription factor 3 (ATF3), which has long been associated with tissue trauma. A growing number of studies indicate that ATF3 may actively promote neuronal axon regrowth and regeneration in species ranging from lampreys to mammals. Here, we review data on the structural and functional conservation of ATF3 protein across species. Comparing RNA expression data across species that exhibit different abilities to regenerate their nervous system following traumatic nerve injury reveals that ATF3 is consistently induced in neurons within the first few days after injury. Genetic deletion or knockdown of ATF3 expression has been shown in mouse and zebrafish, respectively, to reduce axon regeneration, while inducing ATF3 promotes axon sprouting, regrowth, or regeneration. Thus, we propose that ATF3 may be an evolutionarily conserved regulator of neuronal regeneration. Identifying downstream effectors of ATF3 will be a critical next step in understanding the molecular basis of vertebrate CNS regeneration.

## Introduction

While traumatic injury to the mammalian central nervous system (CNS) leads to permanent loss of sensory and motor function, many invertebrate and non-mammalian vertebrate species exhibit a remarkable ability to regenerate nervous system structures and recover functionality. In vertebrates ranging from lampreys and bony fishes to salamanders and reptiles, damage to the nervous system initially triggers loss of function, which is subsequently followed by spontaneous regeneration of severed axons across the lesion site, sprouting of new axon collaterals, and synapse regeneration, ultimately leading to functional recovery of behaviors (Tanaka and Ferretti, 2009; Diaz Quiroz and Echeverri, 2013; Bloom, 2014; Rasmussen and Sagasti, 2016; Morgan, 2017). Even in mammals where spontaneous regeneration of the CNS is notoriously poor, the peripheral nervous system (PNS) undergoes robust regeneration after traumatic injury (Scheib and Hoke, 2013; Cattin and Lloyd, 2016; Gordon, 2020) and selective populations of CNS neurons may have the capacity to activate pro-regernative molecular responses (Matson et al., 2021). Remarkably, when peripheral nerves are used to bridge spinal cord lesions in mammals, this results in a more conducive environment in which CNS axons in the spinal cord can now regenerate (David and Aguayo, 1981; Fawcett, 2018). Thus, neural regeneration is widespread throughout the animal kingdom, suggesting that there must be some conserved molecular mechanisms.

The large number of regenerative animal models, combined with the high degree of conservation across vertebrate genomes, has prompted a search for common molecular pathways that promote successful neural regeneration across species. Indeed, next generation sequencing revealed a set of “regeneration-associated genes” (RAGs) that are intrinsically expressed within neurons and associated with successful regeneration of mammalian PNS axons, as well as CNS axons in many highly regenerative species (Ma and Willis, 2015; Fawcett and Verhaagen, 2018). Amongst the RAGs are several conserved transcription factors that activate or de-activate large sets of genes, placing them as hub proteins in a transcriptional regulatory network induced by injury (Chandran et al., 2016). These include activating transcription factor 3 (ATF3) and AP-1 (Fos/Jun), as well as Sox11, KLF7, and STAT3 (Moore and Goldberg, 2011; Blackmore et al., 2012; Fagoe et al., 2014; Chandran et al., 2016; Mehta et al., 2016; Fawcett and Verhaagen, 2018; Herman et al., 2018). Given their positions as hubs within the injury-induced gene networks, these transcription factors have potential for being master regulators of neural regeneration, and possibly therapeutic targets.

**FIGURE 1:**
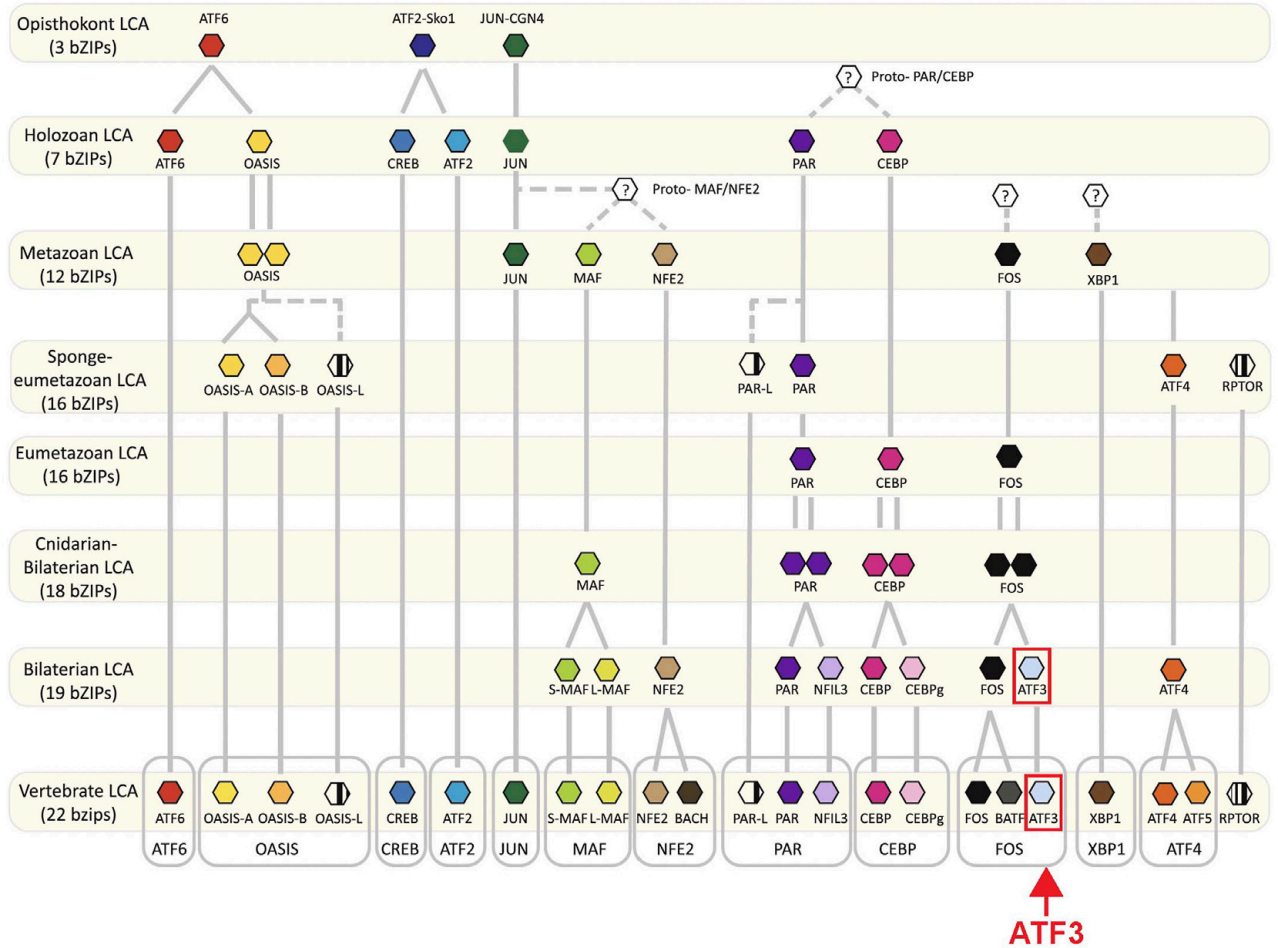
Evolution of bZip transcription factors. Proposed evolutionary timeline of ATF3 and other bZip family members depicts the independent origins of different ATF proteins. The FOS-ATF3 subfamily is highlighted. Adapted from Jindrich and Degnan, 2016, and used with permission as stated under Creative Commons Attribution 4.0 International License https://creativecommons.org/licenses/by/4.0/.

One transcription factor that is emerging as a highly conserved and thus a potentially critical pro-regenerative component for neuronal regeneration is ATF3. ATF3 is a member of the basic leucine zipper (bZip) family of transcription factors (Figure 1). ATF3 diverged relatively late in evolutionary history, having likely evolved from a gene duplication of FOS that occurred before the cnidarian-bilaterian divergence (Figure 1) (Jindrich and Degnan, 2016). In rodents and human cell lines, ATF3 is rapidly induced in response to traumatic injury or cellular stress in a number of tissues including liver, heart, kidney and nervous system, implicating ATF3 induction as part of a general stress response (Liang et al., 1996; Hai et al., 1999). After traumatic injury to the nervous system, ATF3 induction has been observed within the neurons of many diverse vertebrates, including lamprey, zebrafish, and rodents, indicating that this is a highly conserved response (Tsujino et al., 2000; Hui et al., 2014; Chandran et al., 2016; Wang et al., 2017; Herman et al., 2018). Induction of ATF3 and its downstream targets may therefore represent a common molecular pathway that promotes successful neural regeneration across species. In addition, in the non-mammalian CNS and the mammalian PNS, which have robust regenerative potential, ATF3 is amongst the most highly induced RAGs after traumatic injury, making it of particular interest as a potential target (Stam et al., 2007; Hui et al., 2014; Herman et al., 2018; Ewan et al., 2021). The goal of this review is therefore to synthesize the current evidence for ATF3 as a conserved pro-regenerative factor, to explore our current understanding of how it might be working with other RAGs to activate gene transcription, leading to axonal regrowth, and to discuss its potential value as a therapeutic strategy for promoting CNS regeneration after traumatic spinal cord injury (SCI).

### ATF3 Protein is Conserved Across Vertebrates

**FIGURE 2:**
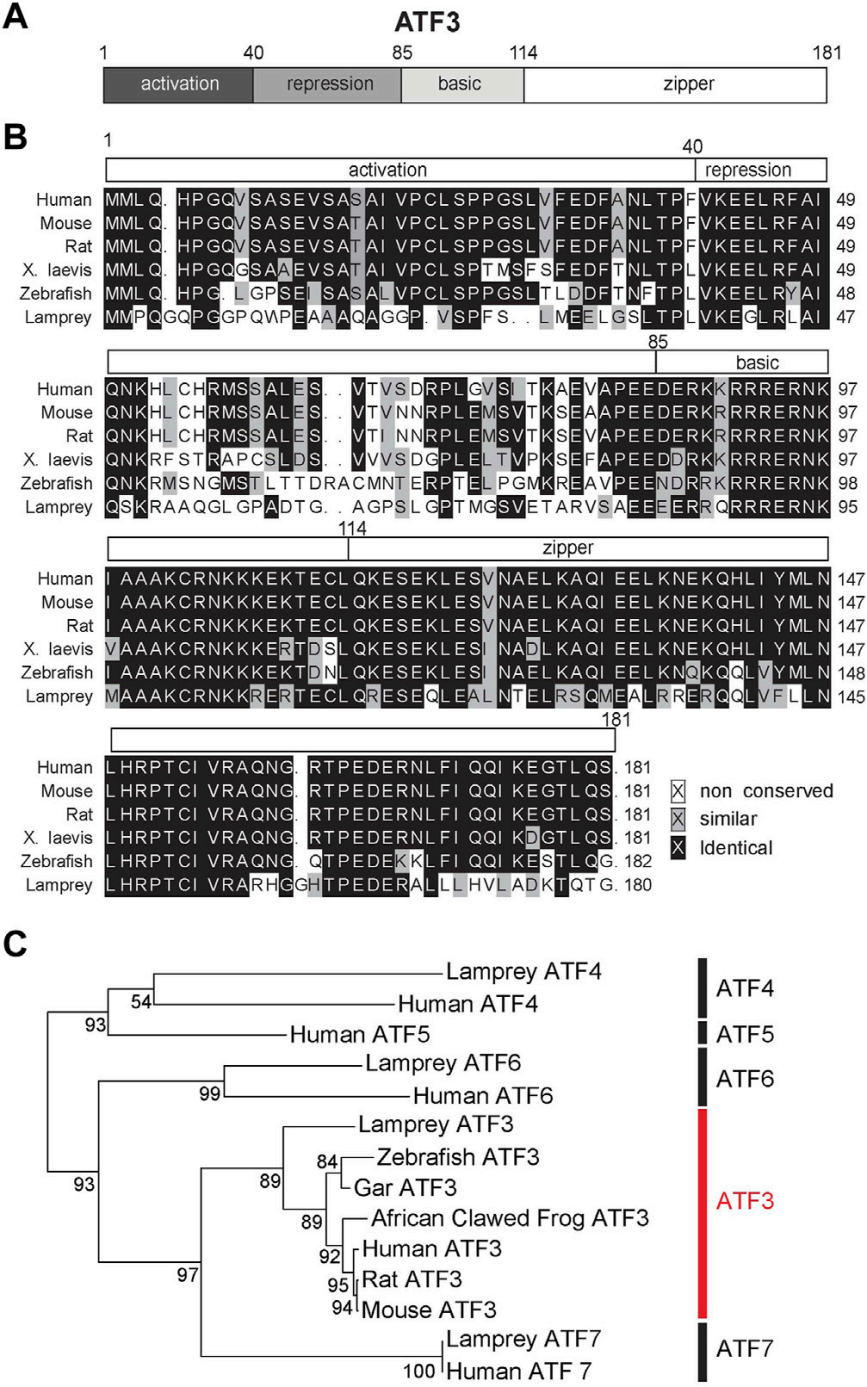
ATF3 is highly conserved from lampreys to humans. **(A)** Domain structure of ATF3. **(B)** Multiple sequence alignment of ATF3 protein. The alignment shows high conservation across model vertebrate species, particularly in the DNA binding basic/leucine zipper (bZip) region (amino acids 85–181). See Table 1 for NCBI Accession Numbers. **(C)** Maximum likelihood molecular phylogeny of several ATF family members, including the lamprey orthologs. Bootstrap values are indicated at nodes. ATF3 subfamily is highlighted in red. Generated in R (version 4.0.2) using the “ape” package.

ATF3 is a 21 kDa protein that contains four distinct regions, including the activation, repression, basic and leucine-zipper domains (Figure 2A). The bZip region of the protein forms the DNA binding domain that is common to ATF/CREB family members (Liang et al., 1996; Jindrich and Degnan, 2016). ATF3 can only bind to DNA as a dimer, and it can homodimerize with itself or heterodimerize with other members of the bZip family of transcription factors, including JUN, FOS, and ATF4 (Rodriguez-Martinez et al., 2017). As a homodimer, ATF3 acts as a transcriptional repressor, but as a heterodimer, ATF3 can act either as an activator or repressor (Hai et al., 1999; Danzi et al., 2018). The specific downstream DNA targets of ATF3 thus vary depending on its dimerization partner (Hai and Curran, 1991; Rodriguez-Martinez et al., 2017), and therefore ATF3 has the potential to impact many downstream pathways.

The primary amino acid sequence of ATF3 shows a high degree of conservation, when compared across vertebrate species such as human (*Homo sapiens*), mouse (*Mus musculus*)*,* rat (*Ratticus norvalus*), African clawed frog (*Xenopus laevis*), and zebrafish (*Danio rerio*) (Figure 2B)*.* To extend this comparison, we also included the ATF3 sequence from sea lamprey (*Petromyzon marinus*), which is amongst the oldest living vertebrate species that evolved from a common chordate ancestor over 550 million years ago (Smith et al., 2013; Herman et al., 2018). Across the vertebrate ATF3 orthologs, the activation, repression, basic, and leucine-zipper domains can all be distinguished, although activation and repression domains are less homologous compared to the highly conserved bZip regions (Figure 2B). When compared to human ATF3, other ATF3 orthologs range from 51% identity (67% similarity) in lamprey to 95% identity (98–99% similarity) in rodents (Table 1). The bZip region of ATF3 (a.a. 85–181) is 62% identical and 83% similar between lamprey and human ATF3, as expected since this sequence is conserved across all bZip family proteins (Jindrich and Degnan, 2016). Phylogenetic analysis including other ATF family members confirms that the annotated sequence in the lamprey genome is indeed an ATF3 ortholog (Figure 2C). Thus, ATF3 is highly conserved amongst vertebrate species, suggesting that it may share similar functions in the nervous system.

**TABLE 1:** Comparisons of vertebrate ATF3 orthologs to human ATF3. Protein-protein BLAST results comparing the ATF3 sequence in each species to human ATF3. NCBI Accession numbers are indicated. ATF3 is highly conserved across vertebrates.

Species	Accession Number	%Identity	%Similarity
*Homo sapiens*	NP_001665.1	100	100
*Mus musculus*	NP_031524.2	95	98
*Ratticus norvegicus*	NP_037044.1	95	99
*Xenopus laevis*	NP_001087487.1	81	88
*Danio rerio*	NP_957258.1	71	81
*Petromyzon marinus*	PMZ_0021004-RA	51	67

### ATF3 is Induced in the Nervous Systems of Highly Regenerative Species Following Traumatic Injury

**FIGURE 3:**
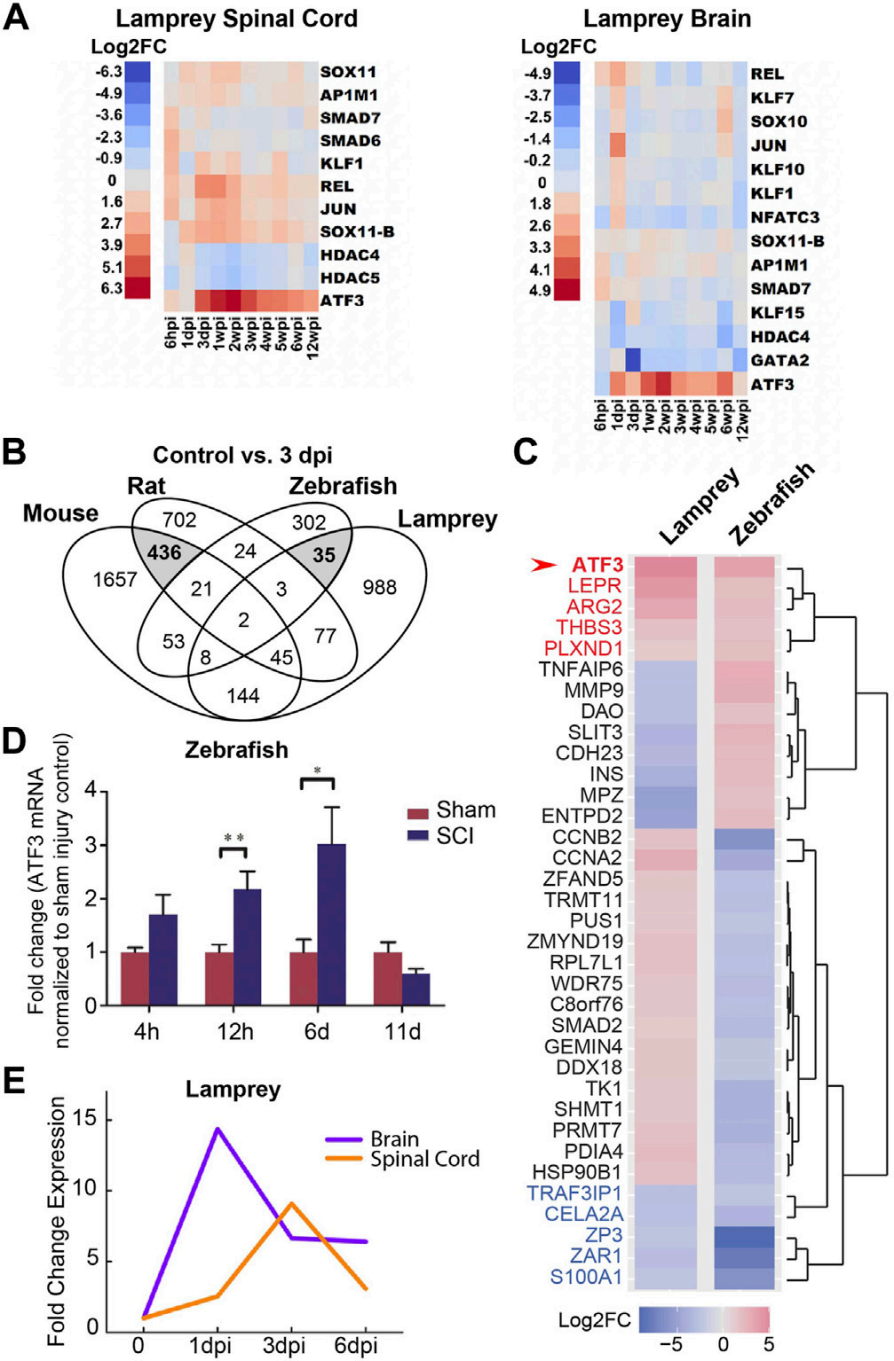
ATF3 mRNA is highly induced after spinal cord injury in zebrafish and lampreys. **(A)** RNA-Seq data shows ATF3 as the most robustly and highly induced RAG in lamprey spinal cord and brain after SCI. **(B)** Venn diagram showing the number of differentially-expressed (DE) genes at 3 days post-injury (dpi) in non-regenerative species (Mouse and Rat) versus regenerative species (Zebrafish and Lamprey). Mouse and rat share in common 436 DE genes at 3 dpi, while zebrafish and lamprey share 35 DE genes (grey). **(C)** Heatmap showing log2 fold change in expression for the 35 DE genes shared between lamprey and zebrafish at 3 dpi. ATF3 was the most highly induced gene in both species (arrow). Red and blue labels indicate genes that were upregulated or downregulated, respectively, in both species. **(D)** ATF3 is highly upregulated after spinal cord injury in zebrafish. Hours (h) and days (d) post-injury are indicated. **(E)** ATF3 is also induced in the lamprey CNS after spinal cord injury. Days (d) and weeks (w) post-injury are indicated. (Panel D reprinted from Wang et al., 2017 *Biochemical and Biophysical Research Communications* 488:522–527, with permission from Elsevier. Panels **(A)** and **(E)** reprinted from Herman et al., 2018 *Scientific Reports* 8:742, and used with permission as stated under Creative Commons Attribution 4.0 International License https://creativecommons.org/licenses/by/4.0/.)

Growing evidence suggests that early induction of ATF3 may be a critical part of the pro-regenerative response after traumatic injury, specifically within nervous system tissues and neuronal cell types that regrow or regenerate their axons. Genome-wide transcriptome and microarray studies have reported that ATF3 is amongst the transcription factors that are most highly induced around the injury site in zebrafish spinal cord after a crush injury (Hui et al., 2014) and in lamprey nervous system after spinal cord transection (Herman et al., 2018). In both species, ATF3 changes from almost undetectable levels to highly-expressed within the first day post-injury and remains high throughout the regeneration period, which includes functional recovery of swimming behaviors (Hui et al., 2014; Herman et al., 2018). In lamprey, ATF3 is strikingly the most highly-induced gene amongst the identified RAGs in both the spinal cord and the brain after SCI (Figure 3A). In contrast, ATF3 induction does not readily occur around the injury site in mouse or rat spinal cord after contusion or compression (Chamankhah et al., 2013; Wu et al., 2013; Sasagawa et al., 2016). Using the data reported in these published studies, we performed a cross-species comparison of the gene expression changes that occurred after SCI in rat (Chamankhah et al., 2013), mouse (Wu et al., 2013), zebrafish (Hui et al., 2014), and lamprey (Herman et al., 2018) at 3 days post-injury, a time point that was reported in all four species. For context, adult zebrafish exhibit a strong proliferative response in the spinal cord by 3 days post-injury, followed by axon regeneration starting around 2 weeks post-injury and behavioral recovery around 4–6 weeks post-injury (Becker and Becker, 2008; Hui et al., 2014; Cigliola et al., 2020). Lampreys follow the same progression, but over a time course of 2–3 months, with proliferation beginning around 1 week post-injury, axon regeneration occurring after 4 weeks post-injury, and behavioral recovery returning by 8–10 weeks post-injury (Rovainen, 1976; Selzer, 1978; Cohen et al., 1986; Oliphint et al., 2010). Rats and mice have somewhat different cellular responses to injury and may regain some reflexes within 7–10 days, but never recover control of voluntary movement (Steward et al., 1999). Although the time course of injury responses and regeneration does differ between zebrafish, lamprey, mouse and rat, having an early post-injury time point in common provides at least a starting point for cross-species comparisons. At 3 days post-injury in mouse and rat, there were 436 differentially-expressed (DE) genes in common between these two non-regenerative spinal cords (Figure 3B). As shown by other studies, amongst the shared DE genes were those associated with inflammation and integrin signalling (Supplementary Table S1). However, ATF3 was not induced in mouse and rat spinal cord at 3 days post-injury (Supplementary Table S1). In comparison, in the highly regenerative zebrafish and lamprey spinal cords, there were 35 DE genes in common at 3 days post-injury (Figure 3B; Supplementary Table S1). Of those, ATF3 was the most highly induced DE gene in both species, suggesting a positive role in spinal cord tissue regeneration, potentially across multiple cell types (Figure 3C). In zebrafish, both microarray and qPCR data showed that ATF3 mRNA expression within the spinal cord increases dramatically within the first few days to weeks post-injury and then gradually declines during the regenerative and functional recovery period (Figure 3D) (Hui et al., 2014; Wang et al., 2017). In the lamprey, unbiased genome-wide transcriptome analysis and qPCR also showed a rapid induction of ATF3 expression in both spinal cord and brain that declined during the regeneration period (Figures 3A,E), implicating a potential role for this gene in supporting pro-regenerative responses both locally within the damaged spinal cord and at supraspinal locations (Herman et al., 2018). Moreover, ATF3 is also rapidly induced after optic nerve crush in zebrafish and is amongst a set of transcription factors with enriched open chromatin binding sites, indicating that active transcription was occurring (Dhara et al., 2019). The rapid and robust induction of ATF3 that follows the recovery period in these systems suggests that ATF3 may activate a new transcriptional program or different functional state of the nervous system, as has been suggested in other contexts ranging from cellular homeostasis and cancer to immune responses (Hai et al., 2010; Ku and Cheng, 2020).

There are also a number of other injury conditions where ATF3 is upregulated in the mammalian PNS and CNS. ATF3 is induced in rodents following injury to peripheral nerves, which are also capable of regeneration. This has now been demonstrated in rat sciatic nerve neurons (Seijffers et al., 2006) and cranio-facial nerve (Gey et al., 2016), as well as cultured dorsal root ganglion (DRG) neurons (Seijffers et al., 2007; Chandran et al., 2016). ATF3 mRNA expression is induced within hours following peripheral nerve injury in rodents and gradually decreases over time (Tsujino et al., 2000; Gey et al., 2016). Interestingly, both CNS lesions and peripheral nerve injury induce an upregulation of ATF3 within DRG neurons (Huang et al., 2006; Stam et al., 2007; Ewan et al., 2021). However, while peripheral nerve injury correlates with an enhanced growth state of DRG neurons (Seijffers et al., 2006; Seijffers et al., 2007; Chandran et al., 2016; Ewan et al., 2021), SCI does not translate into an enhanced growth state of the majority of ascending sensory neurons, perhaps due to the unique downregulation of fatty acid metabolism genes or other distinct transcriptional pathways occurring in the CNS (Stam et al., 2007; Ewan et al., 2021). However, there may some rare neuronal populations in mice that induce expression of ATF3 after SCI (Matson et al., 2021). Moreover, in some axotomized DRG sensory neurons, ATF3 mRNA expression remains atypically elevated several months post-injury (Tsujino et al., 2000; Rau et al., 2016), which is associated with increased sensitization that may contribute to pain (Rau et al., 2016). Several studies have also reported a robust induction of ATF3 and several other RAGs (e.g., c-Jun) in corticospinal neurons after traumatic brain injury in rodents (Mason et al., 2003; Greer et al., 2011; Forstner et al., 2018), but not after a distal injury in the cervical spinal cord (Mason et al., 2003). In one case, subsequent collateral axon sprouting was observed, suggesting that ATF3 induction was also associated with a regenerative response in the brain (Greer et al., 2011). Collectively, these studies reveal that robust, and perhaps temporally-controlled, induction of ATF3 is strongly associated with neuronal regrowth and regeneration in a number of vertebrate species and nervous tissues, but that injury location and other RAGs are likely important factors that determine the robustness of subsequent neural regeneration, perhaps due to the different cellular environments and molecular responses in the CNS versus PNS.

## Evidence for ATF3 as a Neuronal Pro-Regenerative Factor in Neurons

**FIGURE 4:**
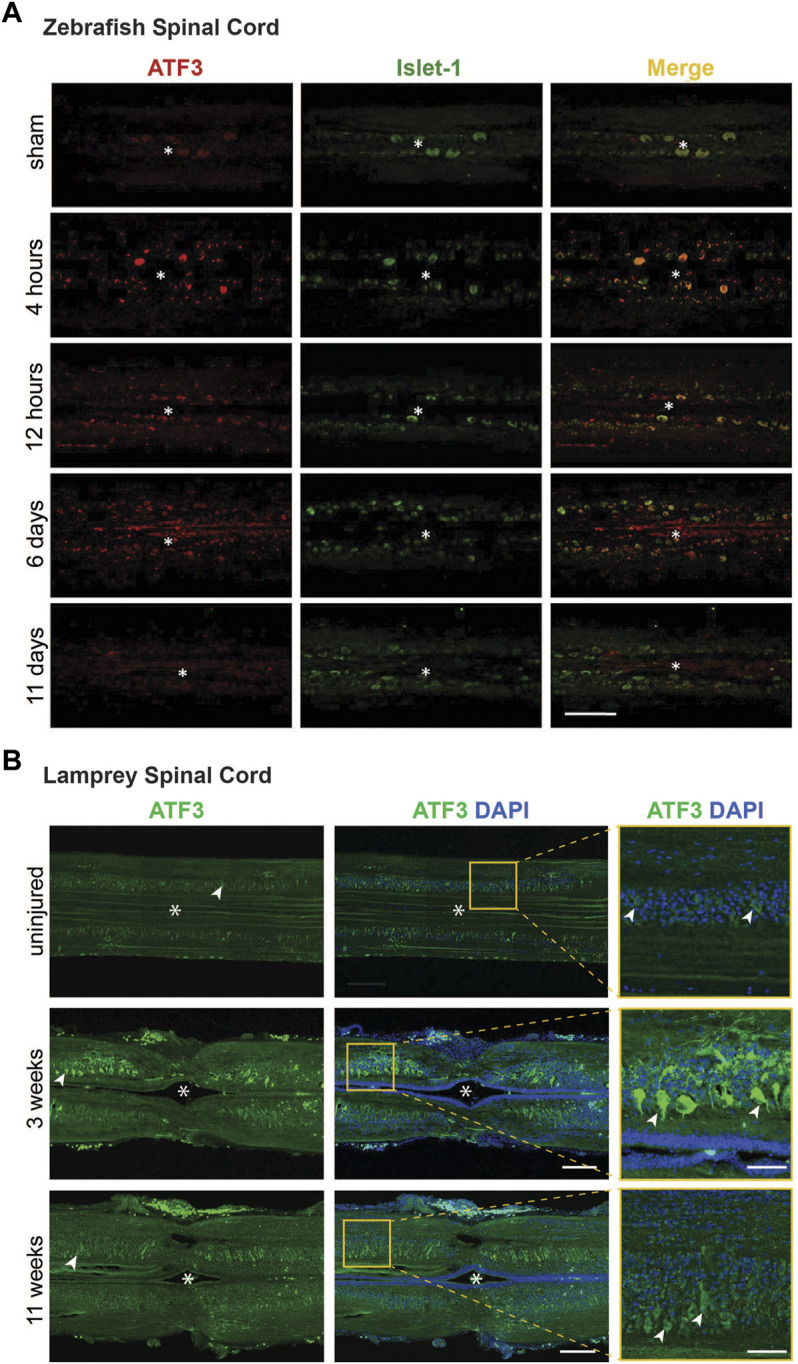
Post-injury ATF3 protein expression in zebrafish and lamprey spinal cord occurs within motor neurons. **(A)** Compared to the sham control, ATF3 protein is upregulated within 4 h after spinal cord injury and steadily declines over the next 11 days. Co-localization with Islet-1 indicates expression in neurons. Asterisks indicate the central canal. Scale bar = 100 μm. **(B)** Similarly, ATF3 is induced in motor neurons within the lamprey spinal cord by 3 weeks post-injury and declines over time. Asterisks indicate the central canal. Arrowheads mark several motor neurons. Scale bar = 150 μm (50 μm in inset). [Panel **(A)** reprinted from Wang et al., 2017 *Biochemical and Biophysical Research Communications* 488:522–527, with permission from Elsevier].

The induction of ATF3 in response to injury of nervous tissues with high regenerative potential is suggestive of a pro-regenerative role. Within the regenerating nervous system, ATF3 expression is primarily localized to neuronal populations, as opposed to glial cells (Gey et al., 2016; Wang et al., 2017; Kole et al., 2020). This is a particularly striking finding, given that ATF3 can be induced in many different tissues and cell types including rodent liver, heart, and macrophages, to name a few (reviewed in) (Hai et al., 1999). Following the injury-induced expression of ATF3 mRNA in zebrafish spinal cord (Figures 3B–D), ATF3 protein levels are also highly upregulated, starting within the first 4 h post-injury and then gradually declining to resting levels around 11 days post-injury (Figure 4A) (Wang et al., 2017). Co-labeling with Islet-1 indicates that the induction of ATF3 protein expression within zebrafish spinal cord occurs in large motor neurons surrounding the injury site, as well as smaller unidentified cells and elongated axonal profiles (Figure 4A) (Wang et al., 2017). In lamprey spinal cord, the post-injury induction and subsequent decline of ATF3 protein expression is also observed in large motor neurons and axonal profiles surrounding the lesion site, though over a longer time period (Figure 4B). To fully understand the extent of ATF3 protein induction, a more detailed examination different cell types is needed, including the descending neurons in the brain which are axotomized by SCI. Similarly, in the mammalian PNS, ATF3 protein induction occurred in neurons of the mouse facial nucleus within the first week after facial nerve injury, peaking at 3 days post-injury (Gey et al., 2016), and in DRG neurons within the first few weeks after sciatic nerve injury (Seijffers et al., 2007). ATF3 protein expression has also been observed in mouse retinal ganglion cells that survive after an optic nerve crush (Kole et al., 2020). Thus, like the mRNA, ATF3 protein appears to be highly upregulated within neurons after injury, again consistent with a positive role for this RAG in neuronal regeneration.

**FIGURE 5:**
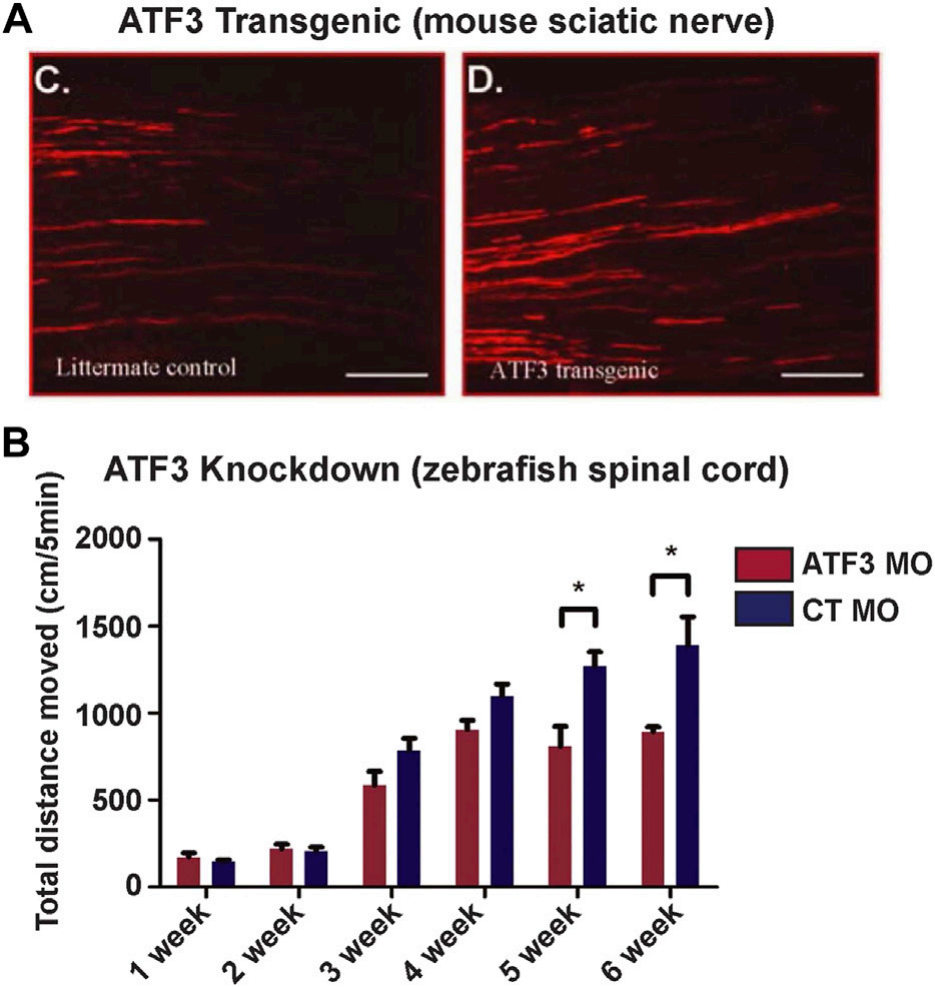
Manipulation of ATF3 influences neuronal regeneration and behavioral recovery. **(A)** Compared to the littermate control, axon regrowth in the sciatic nerve of ATF3 transgenic mice is more extensive after nerve pinch. **(B)** Conversely, ATF3 knockdown with a morpholino (MO) reduced swimming recovery in zebrafish after spinal transection. Weeks post-injury are indicated. [Panel **(A)** reprinted from Seijffers et al., 2007 *Journal of Neuroscience* 27:7,911–7,920, and used with permission. Copyright 2007 *Journal of Neuroscience*. Panel B reprinted from Wang et al., 2017 *Biochemical and Biophysical Research Communications* 488:522–527, with permission from Elsevier].

The current evidence available suggests that post-injury ATF3 induction in neurons promotes greater axonal regrowth, regeneration or sprouting. For example, ATF3 knockdown in the adult zebrafish spinal cord using a translation-blocking morpholino decreased axon regrowth across the injury site at 6 weeks post-injury (Wang et al., 2017). Similarly, ATF3 knockout mice also exhibited decreased facial nerve regeneration compared to wild type mice at 23 and 31 days post-injury (Gey et al., 2016). Conversely, ATF3 overexpression enhanced both peripheral axon regeneration in the mouse sciatic nerve after crush injury (Figure 5A) (Seijffers et al., 2007; Fagoe et al., 2015), as well as regeneration of retinal ganglion cell axons in mouse optic nerve (Kole et al., 2020), but did not improve axon regeneration within the CNS after a spinal dorsal column injury (Seijffers et al., 2007). Enhanced regeneration of DRG axons only occurred when the neurons were cultured on a permissive substrate such as laminin, but not on a non-permissive substrate such as myelin, indicating that ATF3 contributes to the intrinsic growth program of PNS neurons (Seijffers et al., 2007). In zebrafish, ATF3 knockdown in the injured spinal cord not only reduced axon regrowth and regeneration but negatively impacted swimming movements, indicating functional effects of this manipulation (Figure 5B) (Wang et al., 2017). Thus, the limited data available suggest that ATF3 induction promotes axon robust regeneration in highly regenerative models or experimental conditions. However, it remains unclear how ATF3 impacts other aspects of neural regeneration, such as neuronal survival or synapse regeneration, though a recent study in mouse did report that ATF3 overexpression has a neuroprotective effect on a subtype of retinal ganglion cells after optic nerve crush (Kole et al., 2020). Additional studies will be needed in order to fully understand how ATF3 influences regenerative processes beyond its established roles in axon regrowth and regeneration.

## Mechanisms for ATF3-Driven Regeneration

To determine the conditions that are necessary for ATF3 to exhibit a pro-regenerative effect in neurons, it is critical to identify ATF3 dimerization partners and the corresponding gene targets. As mentioned earlier, ATF3 can homodimerize with itself and heterodimerize with many other transcription factors in the bZip family including cJUN, JUNB, and FOS, which then impacts its downstream effectors (Hai and Curran, 1991; Rodriguez-Martinez et al., 2017). ATF3, JUN, and Fos, as well as Myc, RelA, Stat3, Egr1, and Smad1, form a hub of transcription factors in a gene regulatory network that promotes DRG neuron regeneration in the mammalian PNS (Chandran et al., 2016). Although there are likely multiple molecular pathways driving axonal regeneration, which may differ between species and tissues, the over-representation bZip family members within the identified transcription factor hubs suggests an important role for ATF3 and its dimerization partners. Indeed, within the context of neural regeneration, dimerization of ATF3 with cJUN appears to promote greater neurite outgrowth. In both DRG and cortical neurons, co-expression of ATF3 and cJUN promotes significantly greater axon regeneration *in vitro*, compared to either transcription factor alone, suggesting that they work together to promote regeneration in a combinatorial manner (Chandran et al., 2016; Danzi et al., 2018). Further supporting this idea, enhanced regeneration of PNS and CNS axons was also observed after expression of a tethered dimer of Jun ∼ ATF3 (Danzi et al., 2018). Following peripheral nerve injury, a subset of ATF3 expressing neurons also co-express cJUN (Tsujino et al., 2000; Seijffers et al., 2007). However, the upregulation of cJUN appears to be independent of ATF3 overexpression since transgenic ATF3+ mice did not show an equivalent robust increase in global cJUN expression (Seijffers et al., 2007). Thus, ATF3 dimerization with cJUN seems to play an important role in promoting regeneration of some neuronal subtypes.

The cJun-ATF3 dimer activates gene transcription by binding to TRE (AP-1), CRE, and degenerated CRE motifs (Hai and Curran, 1991; Hsu et al., 1992; Rodriguez-Martinez et al., 2017; Danzi et al., 2018). A few studies have begun to explore the downstream effects of ATF3 in the context of neural regeneration. ATF3 seems to suppress pro-inflammatory cytokine response after SCI in zebrafish, since ATF3 knockdown resulted in an increase in TNF-α and IL-1β expression (Wang et al., 2017). This supports previous observations that ATF3 may reduce the acute inflammatory response, which might contribute to its pro-regenerative impact in the nervous system (Jadhav and Zhang, 2017; Forstner et al., 2018). After facial nerve injury in mouse, ATF3 appears to activate a transcriptional network of neuropeptide genes, including *Galanin* and *Grp* whose promoters were also identified as direct ATF3 binding targets (Gey et al., 2016). ATF3 may therefore promote regeneration by reducing acute inflammation and increasing neuropeptide signalling in the injured nervous system.

## Discussion and Future Directions

The search for conserved molecular pathways that promote neuronal regeneration has led to the identification of ATF3 as a potentially critical component. This review highlights its consistent induction during nervous system regeneration across a wide array of vertebrate species, tissue types, and injury models. In addition, ATF3 consistently stands out for its very robust early and prolonged transcriptional induction and protein expression (Hui et al., 2014; Chandran et al., 2016; Wang et al., 2017; Herman et al., 2018), coupled with positive effects on neural regeneration and behavior (Seijffers et al., 2006; Seijffers et al., 2007; Gey et al., 2016). Going forward, it will be important to study how ATF3 affects other aspects of regeneration, including its impacts on neuronal survival, other types of neuronal plasticity (e.g., collateral sprouting), and synapse regeneration, in order to determine whether ATF3 acts as a pro-regenerative switch that turns on all of the above processes. In addition, it will be important to understand how ATF3-driven regenerative processes intersect with other known pro-regeneration pathways, including PTEN/mTOR and cAMP signalling, KLFs, and other regeneration-associated genes that enhance intrinsic growth in neurons (Yang and Yang, 2012; Siddiq and Hannila, 2015; Batty et al., 2017; Williams et al., 2020). It will also be important to explore the identify of rare neuronal populations in mammals that upregulate ATF3 and other RAGs after injury (Matson et al., 2021). It may also be beneficial to test the roles of ATF3 in other *in vivo* mammalian models with unusually high regenerative potential, such as the African spiny mouse (Seifert et al., 2012; Maden and Varholick, 2020) and reindeer antler (Nieto-Diaz et al., 2012), where entire tissues including nervous system must be regrown.

With this review, we provide a rationale for continuing to examine ATF3 induction and its positive roles in enhancing axonal regrowth as a potential strategy for improving neural regeneration in the vertebrate nervous system. However, since ATF3 is constitutively expressed in many non-neuronal cells and tissues and has many different roles in the body, as any potential therapeutic target, it will be important to carefully consider the normal functions of ATF3, the possible downstream effects of manipulating this transcription factor, and possible routes of administration, should this idea move forward in preclinical studies. It will also be critical to identify which of ATF3’s binding partners and potential targets are driving its pro-regenerative role in the nervous system. This may be particularly challenging as ATF3 and its co-activators and repressors are so promiscuous in their binding targets. However, the *in vitro* studies suggest that the pre-dimerized cJUN-ATF3 complex may be a viable tool for promoting neural regeneration that could be developed further for preclinical testing. Although the complexity of the bZip interactions highlights the need for a wholistic approach when considering therapeutic targets for SCI and other conditions where the nervous system is compromised, ATF3 has nonetheless emerged as a promising candidate.

## Supplementary Material

Supplementary Table S1 List of DE genes from mouse, rat, zebrafish and lamprey spinal cord at 3 dpi and the species comparisons. Data correspond to Figure 3A.

Supplementary Table S2 List of 35 DE genes and their log2 fold expression changes in lamprey and zebrafish spinal cord at 3 dpi. Data correspond to Figure 3B.
